# Galactic Cosmic Radiation Induces Persistent Epigenome Alterations Relevant to Human Lung Cancer

**DOI:** 10.1038/s41598-018-24755-8

**Published:** 2018-04-30

**Authors:** E. M. Kennedy, D. R. Powell, Z. Li, J. S. K. Bell, B. G. Barwick, H. Feng, M. R. McCrary, B. Dwivedi, J. Kowalski, W. S. Dynan, K. N. Conneely, P. M. Vertino

**Affiliations:** 1 Graduate Program in Genetics and Molecular Biology, Emory University, Atlanta, GA 30322 USA; 2 Department of Human Genetics, Emory University School of Medicine, Atlanta, GA 30322 USA; 3 Department of Radiation Oncology, Emory University School of Medicine, Atlanta, GA 30322 USA; 4 Department of Biochemistry, Emory University School of Medicine, Atlanta, GA 30322 USA; 5 Department of Biostatistics and Bioinformatics, Rollins School of Public Health, Emory University, Atlanta, GA 30322 USA; 6 Winship Cancer Institute of Emory University, Atlanta, GA 30322 USA; 7 Present Address: Department of Medicine, University of Chicago, Chicago, IL USA

## Abstract

Human deep space and planetary travel is limited by uncertainties regarding the health risks associated with exposure to galactic cosmic radiation (GCR), and in particular the high linear energy transfer (LET), heavy ion component. Here we assessed the impact of two high-LET ions ^56^Fe and ^28^Si, and low-LET X rays on genome-wide methylation patterns in human bronchial epithelial cells. We found that all three radiation types induced rapid and stable changes in DNA methylation but at distinct subsets of CpG sites affecting different chromatin compartments. The ^56^Fe ions induced mostly hypermethylation, and primarily affected sites in open chromatin regions including enhancers, promoters and the edges (“shores”) of CpG islands. The ^28^Si ion-exposure had mixed effects, inducing both hyper and hypomethylation and affecting sites in more repressed heterochromatic environments, whereas X rays induced mostly hypomethylation, primarily at sites in gene bodies and intergenic regions. Significantly, the methylation status of ^56^Fe ion sensitive sites, but not those affected by X ray or ^28^Si ions, discriminated tumor from normal tissue for human lung adenocarcinomas and squamous cell carcinomas. Thus, high-LET radiation exposure leaves a lasting imprint on the epigenome, and affects sites relevant to human lung cancer. These methylation signatures may prove useful in monitoring the cumulative biological impact and associated cancer risks encountered by astronauts in deep space.

## Introduction

The potential for human interplanetary travel and deep space excursion are currently limited by concerns surrounding the long–term human health risks associated with galactic cosmic ray (GCR) exposure^1–4^. These risks include degenerative effects on the cardiovascular and central nervous systems and the risk of cancer at sites such as the lung, colon, breast, and stomach^5^. Given the absence of direct epidemiologic data, GCR exposure risk estimates currently rely on modeling based on data from ground-based experiments with cells and animals.

Terrestrial radiation is composed primarily of low linear energy transfer (low-LET) photons (e.g. γ-rays or X rays) that are sparsely ionizing and deposit energy in a dispersed manner in tissue. In contrast, the GCR spectrum is composed of hydrogen, helium and heavier atomic nuclei with high charge and energy (HZE), including ^28^Si, ^56^Fe, and other ions. Though a low fraction overall, these heavy particles are of particular concern as they have high linear energy transfer (high-LET) values and leave a concentrated track composed of a densely ionizing, nanometer-scale core and a penumbra of high-energy secondary electrons (δ rays) that can extend laterally for several microns as they traverse tissue^3,6^. This creates a tightly clustered and complex mixture of DNA damage (double strand breaks, single strand breaks, base damage, etc.), which is a challenge to repair^4–7^. GCR also generates non-targeted effects in cells not directly traversed by radiation tracks (bystander effects), which may account for as much as half of the cancer risk at doses relevant to human exposure^8^. The unique biophysical properties of high-LET ions are also being exploited as a novel modality for cancer radiotherapy where the opportunity to deliver dense ionization selectively within the tumor volume has the potential to increase the efficacy for tumor control while minimizing normal tissue toxicity. Indeed, carbon-ion beam therapy is currently being evaluated for the treatment of brain tumors and other cancer types in Europe and Asia^9^. A better understanding of the biological effects of HZE particle exposure therefore has important implications for cancer causation and its treatment.

The different heavy ions that make up the GCR spectrum each have distinct effects on gene expression patterns in cultured cells, via mechanisms that remain poorly understood^10^. These differences in gene expression may reflect modifications to the epigenome. Unlike the underlying DNA sequence, the epigenome, collectively represented in the local patterns of DNA cytosine methylation, posttranslational modifications of histones, nucleosome positioning, and long-range chromatin organization, can change readily over time and may represent an important feature of how organisms adapt to a changing environment^11,12^. In particular, DNA methylation, which occurs primarily at cytosines (5mC) in the context CG (CpG), is propagated at each cell division by the action of the DNMT1-UHRF1 complex, which copies the methylation status of CpGs on the parental DNA strand to the newly-synthesized strand, a specificity imparted by a preference for hemi-methylated CpG dinucleotides^13^. Therefore, induced changes in the DNA methylation patterns have the potential to persist over multiple cell divisions, resulting in a lasting and mitotically heritable “memory” of prior exposures. Once considered to be a fairly permanent mark, recent work indicates that DNA methylation in some regions can be dynamically remodeled through TET-mediated oxidation of 5mC to 5-hydroxymethyl cytosine (5hmC), 5-formylC (5fC), and ultimately 5-carboxyC (5caC). Subsequent removal of 5fC and 5caC by the base excision repair machinery results in a net “demethylation” (reviewed in^14,15^). Such induced alterations to the epigenome, in addition to changes to the genome, have the potential to contribute to altered gene expression programs and the long-term consequences of radiation exposure.

Research addressing the effects of radiation exposure on the epigenome have revealed complex relationships, dependent on the radiation source, the model system utilized (whole body vs. tissue culture cells), and the genomic fraction analyzed (eg. global levels of 5mC, the methylation status of certain repetitive elements vs. gene/site-specific analyses)^16–34^. In general, exposure to low-LET X-irradiation has been associated with global or repetitive element hypomethylation^16,17,19,30–32,35^, although gene- and CpG site- specific hypermethylation^19,36,37^ and hypomethylation^38^ have also been observed, and the specific effects exhibit tissue, gender, and strain-specificity^19,20,31,37,39^. In some cases, these alterations are accompanied by altered levels of DNMTs, methylated DNA binding proteins, or the activation of miRNAs suggested to target DNMTs and other chromatin modifiers^30,32,35,36,38^. In contrast, exposure to protons and high-LET species (^28^Si, ^56^Fe, ^48^Ti-ion) are often, though not always, associated with increases in global and/or repetitive element methylation^17,18,21,22,26,28,29,34,39,40^ although this too exhibits significant dose, time (acute vs. latent) and tissue/cell type dependence^16–18,33,35^. Alterations at select genes have also been observed in response to high-LET ions^33,40^. The finding that changes in repeat element and global DNA methylation levels are more similar between proton- and ^56^Fe-ion exposed than X ray exposed cells suggests that radiation-induced changes in DNA methylation may be more tightly linked to radiation quality than to LET alone^39^. Few studies have directly compared the effects of site-specific DNA methylation changes between low-LET terrestrial radiation (X ray) or among high-LET HZE particle radiation species on a genome-wide scale.

Here we examined the effects of ^56^Fe and ^28^Si, two ions found in GCR, on the methylation status of over 485,000 CpG sites spanning 99% of RefSeq genes and 96% of CpG islands across the human genome. We assessed the acute impact (48 hr. post irradiation) and long-term persistence of DNA methylation changes induced by each exposure and compared that to the effects of X rays, on immortalized human bronchial epithelial cells. We find that dose-dependent changes in DNA methylation are observed early and persist over time, with each insult having unique characteristics with regards to the direction, distribution, and underlying chromatin compartment affected, suggesting that these changes arise through distinct mechanisms and may have distinct biological consequences. Further, we find that the ^56^Fe ion-induced methylation signature uniquely reflects a cancer-specific methylation pattern observed in human primary lung cancers. Together these results speak to an epigenetic ‘memory’ of space radiation exposure.

## Results

The goals of this study were to define the acute impact (48 hrs.) and long-term persistence of radiation exposure on the epigenome, and to directly compare the effects of high-LET GCR components (^56^Fe ion, 170 keV/µm; ^28^Si ion, 70 keV/µm) and low-LET X rays (2 keV/µm). We hypothesized that induced changes to the DNA methylation pattern would provide a lasting imprint of the acute radiation exposure with the potential to contribute to the long-term health risks, including cancer. To test this hypothesis, triplicate cultures of immortalized human bronchial epithelial cells (HBEC-3KT)^41^ were exposed to high-LET radiation (^56^Fe ion: 600 MeV/u at 0, 0.1, 0.3, 1.0 Gy; ^28^Si ion: 300 MeV/u at 0, 0.3, 1.0 Gy) at the Brookhaven National Laboratory NASA Space Radiation Laboratory (NSRL) or to low-LET radiation (X ray: 0, 1.0 Gy) at Emory University. Samples were collected from a fraction of the exposed population after 48 hrs. and the remaining cells were maintained in continuous culture for an additional ~35 population doublings (~2.5 months). Cells were collected for genomic DNA extraction at 48 hrs., and thereafter at ~1 week intervals. Non-irradiated control cultures underwent the same handling procedures (including travel to/from the NSRL) and were maintained in parallel. Triplicate cultures were kept as independent biological replicates throughout the course of the experiment. While irradiation elicited some acute cell death (~40% at the highest doses of ^56^Fe), the majority of cells survived to confluence, and continued to grow at a similar rate of ~0.5–0.6 population doublings per day from that point onward, regardless of treatment group.

### Radiation-induced changes to the epigenome are LET dependent and ion specific

**Figure 1:**
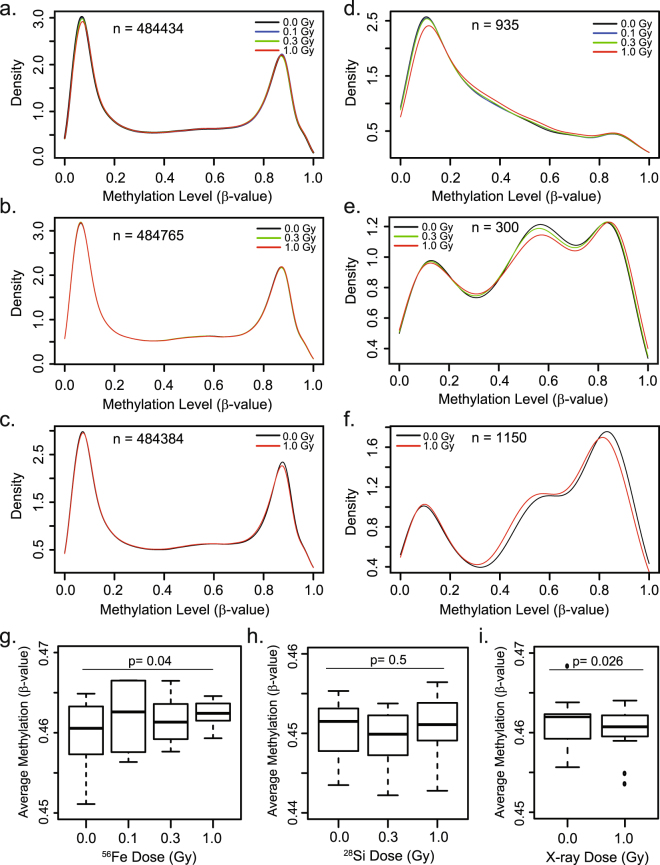
Global impact of high- vs. low- LET radiation on DNA methylation. (**a**–**f**) Density plots showing the impact of the indicated dose of each radiation source on the distribution of DNA methylation values (β values) across all time points, for all evaluable (>484,000 CpG sites on the array passing QC) CpG sites (**a**–**c**) or the subset of CpG sites whose methylation status was found to be significantly associated with increasing dose of ^56^Fe, ^28^Si or X ray exposure (**d**–**f**). (**g**–**i**) For each sample, the average methylation (β value) across all evaluable CpGs (>484,000 CpG sites) was determined. Box plots representing the distribution of the average methylation level across all 12 samples (3 replicates x 4 time points) for the indicated dose/source. The line represents the median average methylation level, boxes the first and third quartiles, whiskers represent the interquantile distance. Note the trend towards hypermethylation with increasing ^56^Fe ion dose (p = 0.04) and towards hypomethylation with X ray dose (p = 0.026). No significant directional trend was observed with ^28^Si exposure. P-values were determined as a regression of the mean beta value across all samples with dose, with covariates for time, batch and array.

The methylation status of 485,577 CpG sites was assessed for each DNA isolate (triplicate samples for each treatment dose and time-in-culture) using the Illumina Infinium HumanMethylation 450 K Platform. DNA methylation levels at each CpG site are represented as a β-value that estimates the percent of methylated alleles at each CpG position in the DNA sample. We found that the effects of irradiation on CpG methylation patterns were dependent on radiation type (^56^Fe ions, ^28^Si ions, X rays; Fig. 1a–c,g–i). When considered globally, there was an average dose-dependent trend towards hypermethylation in response to ^56^Fe ion exposure (Fig. 1a,g), no trend in the global average methylation levels in response to ^28^Si ion exposure (Fig. 1b,h), and an average dose-dependent trend towards hypomethylation in response to X ray exposure (Fig. 1c,i).

For each radiation type, we next applied a linear mixed effects model to identify CpG sites where the levels of methylation changed significantly with dose. This approach allows for an independent assessment of methylation changes significantly associated with radiation dose by accounting for other covariates, such as time-after-exposure. We identified 935 CpG sites where the methylation status was moderately associated with dose of ^56^Fe ions (849 hypermethylated; 86 hypomethylated, p < 0.001); 300 sites where the methylation status was associated with ^28^Si ion dose (158 hypermethylated, 142 hypomethylated, p < 0.001) and 1,150 where the methylation status was associated with X ray dose (252 hypermethylated; 898 hypomethylated, p < 0.001) (Supplemental Table 1). ^56^Fe ion exposure tended to affect CpG sites that are less methylated at baseline (mean = 21.9% methylation) and to induce their hypermethylation (Fig. 1d), whereas ^28^Si ion exposure primarily affected CpG sites that start with intermediate DNA methylation levels (eg. ~40–80% methylated) and had a roughly equivalent tendency to promote their hyper or hypomethylation (Fig. 1e). X ray exposure primarily affected more highly methylated CpG sites (median = 61.9% methylated) and led to their hypomethylation (Fig. 1f).

**Figure 2:**
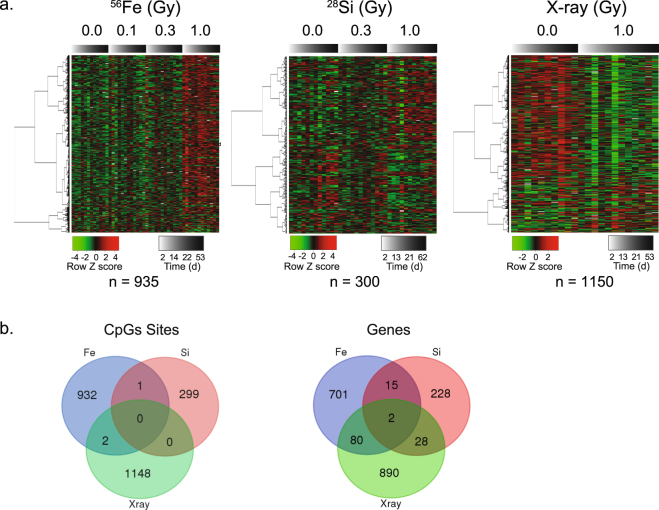
Differential effects of High- (^56^Fe, ^28^Si) or low-LET radiation dose on the methylation status of individual CpG sites. A linear mixed effects model was used to identify DNA methylation changes significantly associated with dose, source, or time-after-exposure (see Methods). This analysis identified 935 CpG sites whose methylation status was moderately (p < 0.001) associated with ^56^Fe dose (849 hyper; 86 hypo); 300 CpG sites associated with ^28^Si dose (158 hyper, 142 hypo) and 1150 CpG sites associated with X ray dose (252 hyper; 898 hypo). (**a**) Heatmap showing the methylation status (low, green to high, red) of the ^56^Fe, ^28^Si or X ray significant CpG sites (rows) for each replicate sample analyzed (columns). Samples (columns) are arranged from left to right by increasing time after exposure (shaded gray bar, n = 3 for each time point) for the indicated dose (n = 12 for each indicated dose; 4 time points in triplicate). (**b**) Overlap between individual CpGs sites (left) or nearest RefSeq gene (right) among CpGs differentially methylated in response to ^56^Fe,^28^Si, X ray. Note the largely distinct CpGs affected by each radiation type.

The same trends were also evident in the analysis of the individual affected sites. A heat map representation of individual CpG sites again showed the preponderance of hypermethylation events for ^56^Fe ion-exposed cells, nearly equivalent hyper and hypomethylation observed for ^28^Si ion-exposed cells, and hypomethylation among X ray exposed cells (Fig. 2a). As implied by the genome-wide trends, the CpG sites affected by each type of radiation exposure showed distinct patterns. Indeed, there was little overlap in the specific CpG sites affected by each radiation type (two or fewer CpG sites shared in total between any two radiation types, Fig. 2b). Taken together, these data are consistent with a graded methylation response with regards to LET (^56^Fe, 170 keV/µm; ^28^Si, 70 keV/µm; X rays, 2 keV/µm) rather than a sharp distinction between high-LET heavy ions and low-LET photon (X ray) irradiation.

### Radiation-induced changes to the epigenome occur early and persist over time

**Figure 3:**
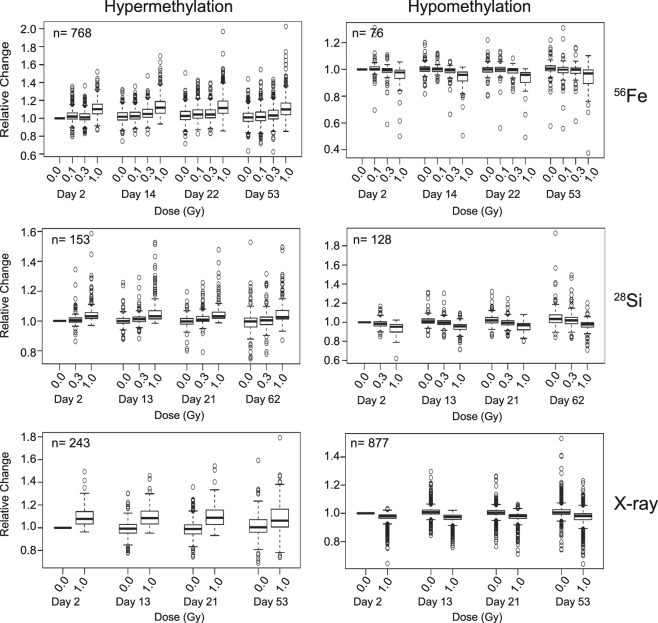
Fate of Radiation-induced changes in DNA methylation over time. CpG sites exhibiting a change in methylation level significantly associated with radiation dose were normalized to their individual initial methylation levels as extrapolated from the 48 h, unexposed cultures. Shown is the distribution of the change in methylation of CpG sites undergoing hyper- or hypo-methylation in response to the indicated radiation source relative to the internal control (unexposed) cultures at 48 h. Line represents the median, boxes the first and third quartile, and whiskers extend to maximum value that is 1.5 times the interquartile range. For clarity, CpG sites whose methylation level was also independently associated with time-after-culture were excluded. Note that irradiation-induced methylation changes occur early (within 48 h) and largely persist over time.

We next considered the fate of radiation-induced DNA methylation changes over time. To focus specifically on the fate of radiation-induced methylation changes, we selected those CpG sites where the change in methylation was moderately associated with dose, but were not also independently associated with time-dependent methylation ‘drift’ (see below). This left 844 ^56^Fe ion-affected CpG sites (768 hyper; 76 hypo), 280 ^28^Si ion-affected sites (153 hyper, 128 hypo) and 1120 X ray-affected sites (243 hyper, 877 hypo). We determined the change in mean β-value over time, relative to non-irradiated control cells at 48 hr (the earliest time point) (Fig. 3). Although there was some variation in methylation with time among non-irradiated cells (note that the distribution of methylation levels at the 0 Gy dose broadens over time in each panel of Fig. 3), the dose-dependent change in DNA methylation induced by each radiation source evident two days after radiation exposure was largely retained more than 50 days later (28–34 population doublings) (Fig. 3). These data suggest that in general the radiation-induced methylation changes occur early and persist over time, resulting in a heritable change in the epigenome.

### Methylation “drift” over time

As noted by others^36^, we observed considerable methylation “drift” over time in cell culture, independent of radiation exposure (Supplemental Fig. 1a,d). Indeed, application of the linear mixed effects model identified thousands of sites significantly associated with time-after-exposure, independent of dose (i.e., when dose was considered as a covariate). For each exposure type, >2,900 sites were significant after Bonferroni (Holm) adjustment (p < 1e-7) and >77,000 CpG sites were significant according to an FDR criterion (FDR < 0.05; p < 0.01). The average rate of change was consistent across the different experimental series performed over two years at different times, and was estimated to be 0.001% methylation per day, or the equivalent of a shift in methylation status of 1 in 1,000 DNA molecules per day. A comparison of those sites significantly associated with time from each experiment indicated that, whereas there was significant overlap in the sites affected from one series to the next, the direction of change was not always the same. Indeed, the two series that were performed in parallel and in the same time-frame (^28^Si ion, X ray) showed the greatest concordance with respect to both the sites affected and direction of change, but were less concordant when either was compared to the ^56^Fe ion series which occurred at a later date (Supplemental Fig. 1b,c). Our results suggest that although many CpG sites are prone to methylation drift in cell culture, other factors appear to impact the direction of drift (i.e., hyper- vs. hypo-methylation). A comparison of those sites whose methylation state was significantly associated with both radiation dose and time (n = 91 for ^56^Fe ion; 19 for ^28^Si ion; and 30 for X ray) showed that the effects of irradiation and intrinsic drift were largely independent in that once imposed, the effect of radiation dose on individual CpGs had little impact on the rate or direction of drift over time (Supplemental Fig. 1d). Thus, the effects of irradiation appear to be superimposed upon an intrinsic tendency for methylation to drift with cell division.

### HZE ions of different charge and energies affect different genomic compartments

**Figure 4:**
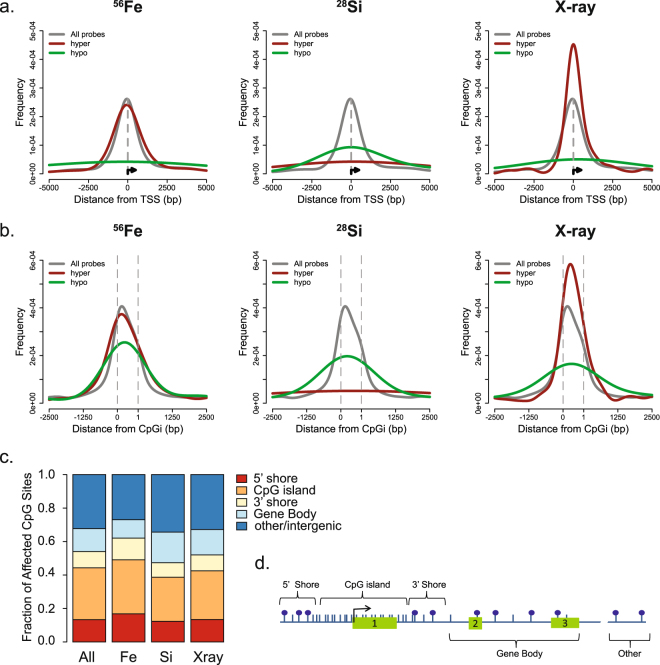
Genomic location of CpG sites significantly associated with radiation dose. (**a**) Average distance of all CpGs on the array (gray), or the subset that underwent hyper (red) or hypo (green) methylation in response to increasing ^56^Fe, ^28^Si or X ray dose relative to the transcription start site (TSS) of the nearest gene oriented to the direction of transcription. (**b**) Average distance of all CpGs on the array (gray), or the subset that underwent hyper (red) or hypo (green) methylation in response to increasing ^56^Fe, ^28^Si or X ray dose relative to the nearest CpG island. The distribution within the CpG island is scaled to size (dotted gray lines), and includes a fixed distance of +/−2.5 kb in either direction from the CpG island edge. (**c**) Fraction of ^56^Fe, ^28^Si or X ray affected CpG sites that lie within the indicated gene compartment relative to that of All CpG sites interrogated on the array. CpG sites were annotated to the nearest CpG island associated RefSeq gene. CpG islands defined by UCSC criteria, 5′ and 3′ shores are 2,000 bp from the 5′ and 3′ CpG island edge. Gene bodies were considered the region from the 3′ edge of the CpG island +2 kb, to the transcription end site (TES). CpG sites not overlapping one of these features were considered to be intergenic/other. (**d**) A schematic of the genomic compartments described in *C*. Shown is a hypothetical gene (exons-green boxes) for which the TSS (black arrow) is embedded in a CpG island promoter. Blue ticks represent CpG sites, blue balls as methylated CpG sites. The TES would be the end of exon 3.

Given the largely independent subsets of CpGs affected by the different radiation types, we next sought to determine the relationship between source-specific DNA methylation changes and other genomic and epigenomic features. We examined the distribution of CpG sites significantly associated with ^56^Fe ion, ^28^Si ion, or X ray dose relative to genetic features, including the distribution in and around CpG islands and genes (Fig. 4). Relative to the distribution of all probes on the array, ^56^Fe ion-affected CpG sites, most of which were hypermethylated, tended to lie within CpG islands (which generally lack DNA methylation) and around transcription start sites (TSS). These hypermethylated sites were particularly enriched in CpG island “shore” regions (defined here as 2 kb from the 5′ or 3′ edge of the CpG island domain), while the few sites that became hypomethylated arise from outside these regions and away from CpG islands. In contrast, ^28^Si ion-affected sites tended to be depleted in CpG islands and shores and instead were enriched among gene bodies and other distal regions. Overall, X ray-affected sites were distributed similarly to the probes on the array. The majority of sites that were hypomethylated were located in genomic regions outside of CpG islands, which are typically methylated; the few sites that were hypermethylated were enriched in CpG islands, which are typically unmethylated.

**Figure 5:**
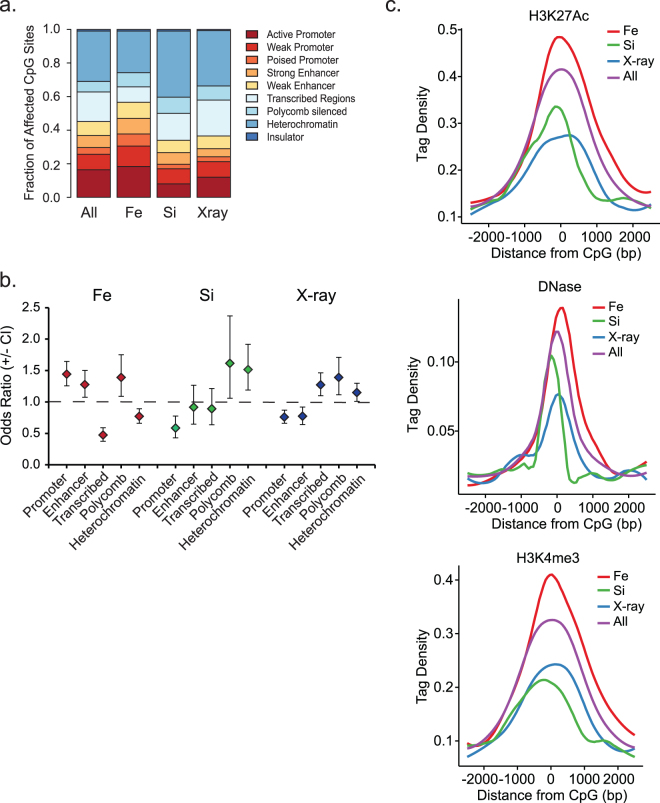
HZE-particles of distinct LETs affect methylation of CpG sites in different genomic chromatin compartments. CpG sites were annotated to a chromatin-based functional genomics annotation, ChromHMM, established by Ernst *et al*.^42^ using 14 different chromatin features from ENCODE data from human epithelial cells (HMEC). Shown is the fraction (**a**) and relative enrichment (**b**) of ^56^Fe, ^28^Si or X ray affected CpG sites that overlap in the indicated compartment, relative to that of ‘All’ CpG sites on the array. Data represent the odds ratios determined by Fishers exact +/− the 95^th^ confidence interval. (**c**) Normalized average tag densities of H3K27 acetylation ChIP-seq (*Top*), DNaseI-seq (*Middle*) or H3K4me3 ChIP-seq (*Bottom*) surrounding all assayed CpGs (All), or the subset of CpGs whose change in methylation was significantly associated with ^56^Fe, ^28^Si or X ray dose. Data are derived from ENCODE CHIP-seq and DNAse-I seq data from A549 lung cancer cells^76^. Note the over-representation of H3K4me3, H3K27ac, and DNaseI accessibility at ^56^Fe-affected sites.

To further investigate the genomic compartments affected by radiation-induced methylation changes, we examined the relationship to chromatin features. Using genome-wide ChIP-seq data for histone modifications, RNA polymerase occupancy, and other chromatin features, Ernst, *et al*.^42^ used a hidden Markov model to partition the genome into functional domains, termed ChromHMM. We analyzed the ChromHMM states and existing genome-wide datasets to evaluate the chromatin structure surrounding the irradiation-sensitive CpG sites. This analysis revealed that the ^56^Fe ion-affected sites were more likely to occur in areas with a more “open” chromatin structure, including promoters and enhancers (Odds Ratio = 1.3–1.5 fold; p < 0.004), but were depleted from the transcribed regions of genes (Odds Ratio = 0.47, p = 2.8E-13; see Fig. 5). Consistent with a propensity for promoters/enhancers, ^56^Fe ion-affected sites were enriched in regions that are accessible to DNase I and marked by acetylated histone H3 lysine 27 (H3K27ac), a mark of active enhancers, relative to all sites on the array and as compared to the ^28^Si ion- or X ray-affected sites, which were depleted in these features. In contrast, ^28^Si ion-affected sites were depleted in genes and features of active/accessible chromatin (i.e. H3K27Ac, DNaseI accessibility, H3K4me3) and were more likely to occur in repressed chromatin environments (i.e. sites marked by heterochromatin and polycomb; Odds Ratio = 1.5–1.6, p < 0.02; Fig. 5). X ray-affected sites were enriched in transcribed regions (Odds Ratio = 1.3, p < 0.001) (consistent with an enrichment in gene bodies shown above), but relatively depleted in features of active/accessible promoters and enhancers.

Taken together, the above data suggest that different sources of radiation preferentially affect sites in different chromatin contexts (e.g. enhancer, promoters, condensed chromatin), which could underlie their distinct biological consequences. To further evaluate the potential functional significance of the radiation-induced changes to the epigenome, we annotated the ^56^Fe, ^28^Si and X ray-affected CpG sites to their nearest RefSeq gene (Supplemental Table 1). Consistent with a largely independent set of CpG sites affected by each radiation type, the genes to which the CpGs mapped were also largely distinct, with fewer than 10% in common between any two radiation types (see Fig. 2b). Gene ontology and gene set enrichment analyses indicated that the genes associated with the ^56^Fe-affected CpGs tended to be enriched in genes involved in transcriptional regulation and development/morphogenesis, whereas the X ray affected sites tended towards genes encoding protein kinases, signal transduction, or stress responses (eg. p53, retinoic acid) (Supplemental Table 2). The ^28^Si class tended towards genes involved in cell signaling and immune related functions. Interestingly, genes associated with the hypermethylated CpGs in the ^56^Fe affected group showed a significant overlap with genes whose promoters were previously shown to undergo DNA hypermethylation in lung cancer cells^43^ (n = 31, FDR corrected p = 3.75 e-12; Fishers Exact), including several homeobox containing transcription factors, the RNA binding protein lin28A, and the chemokine, CXCL12 (Supplemental Table 2).

### Methylation status of ^56^Fe ion-affected CpG sites distinguishes primary lung tumor from normal tissue

**Figure 6:**
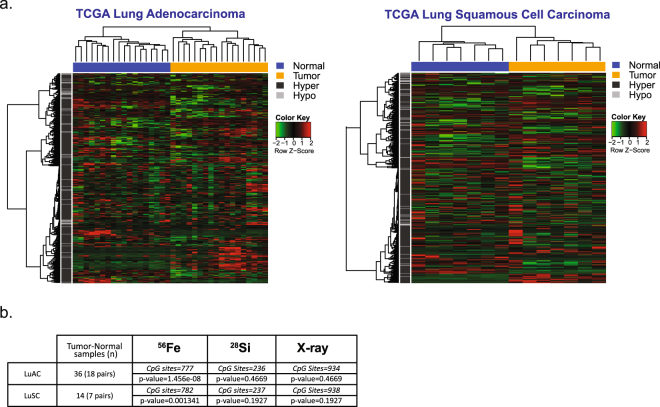
The ^56^Fe-specific methylation ‘signature’ discriminates lung tumor from normal tissue in primary tissue samples. (**a**) DNA methylation status of the CpG sites significantly associated with Fe dose (n = 777) in normal bronchial epithelial cells was extracted for 25 lung tumor-normal pairs (18 adenocarcinomas, 7 squamous cell carcinomas) available in the TCGA project and used in unsupervised hierarchical cluster analysis (complete linkage, Manhattan distance metric). (**b**) An equivalent number of CpGs were chosen at random and used to group tissue samples using the same approach, and the process was repeated 1,000 times to estimate significance. The ^56^Fe-sensitive CpGs outperformed any random set by several orders of magnitude. The methylation status of the ^28^Si or the X ray affected sites had no significant association with tumor-specific differences in methylation (see Methods).

Taken together, the above data indicate that particles of different qualities and energies have unique impacts on the epigenome, which may ultimately manifest in distinct biological consequences. We next sought to determine the relevance of these radiation-induced CpG methylation changes to human lung cancer. We leveraged the human epigenome information available from hundreds of primary lung tumors that have been analyzed on the HumanMethylation450K platform as part of the Cancer Genome Atlas (TCGA) Project. Level 3 DNA methylation data (β-values) were extracted for the ^56^Fe ion- (n = 935), ^28^Si ion- (n = 300) and X ray- (n = 1150) sensitive CpG sites for a set of 18 tumor-normal pairs of human lung adenocarcinoma (LUAC) and 7 tumor-normal pairs of squamous cell carcinoma of the lung (LUSC). The methylation status of these sites was then used in an unsupervised cluster analysis (complete linkage clustering, Manhattan distance). Interestingly, the methylation status of the ^56^Fe ion sites, in particular, cleanly separated primary tumor specimens from normal tissue for both the LUAC samples (p = 1.46e-08) as well as the LUSC samples (p = 0.0013), whereas neither the ^28^Si ion-affected CpG sites nor the X ray- affected CpG sites showed any significant association (Fig. 6). To test the robustness of the separation achieved by the methylation at the ^56^Fe ion-affected sites, the clustering approach was repeated 1,000 times using an equivalent number of CpG sites (n = 777, LUAD; n = 782, LUSC) chosen at random from a total of ~390,000 CpG sites with a detection p-value across all TCGA samples of <0.05. None achieved significance greater than the ^56^Fe ion signature sites for either LUAD (random sampling p-value range 0.0005–0.0850; median = 0.009) or LUSC (random sampling p-value range 0.0590–0.1069; median 0.0885). Thus, the methylation status of CpG sites sensitive to ^56^Fe ion exposure in human bronchial epithelial cells is uniquely characteristic of human lung cancer.

## Discussion

Ionizing radiation (IR), such as γ- or X rays, increases the age-related risk of many common human cancers, with lung cancers representing about a third of cases linked to prior radiation exposure among atomic bomb survivors and occupational exposures in nuclear reactor workers^5,44,45^. In the absence of epidemiologic data on humans exposed to GCR, current estimates of cancer risk are based primarily on animal models, which have shown that exposure to high-LET radiation sources results in a greater tumorigenic potential and a more aggressive phenotype (e.g. shorter latency, accelerated progression, and increased metastatic potential) as compared to low-LET sources^46–50^. However, the degree to which these cancer risk estimates can be directly extrapolated to astronauts and space radiation exposure is fraught with uncertainties, due in part to incomplete understanding of the biological impact of high-LET radiation exposure and how it differs from terrestrial radiation sources as well as other confounding factors such as smoking status^46,51^. Thus, biomarkers that can be used to monitor exposures and reliably predict disease risk are sorely needed.

Here we show that HZE particles induce a unique imprint on the epigenome. Significantly, we found that radiation-induced methylation changes occur early and persist over time, reflecting a heritable change to the epigenome. Impey *et al*.^23,24^ similarly found that alterations in 5mC and 5hmC in the mouse hippocampus observed at 2 weeks after whole body proton irradiation persisted for at least 5 months, whereas only a subset of those induced by low dose (0.2 Gy) ^56^Fe exposure were stable. Likewise, Wang *et al*.^19^ found that while the global hypomethylation after low dose whole body X-irradiation resolved, the hypermethylation of select gene promoters persisted for >1 month. We further find that the radiation-induced changes to DNA methylation were source-dependent, and impact DNA in different chromatin contexts, implying that they arise through distinct mechanisms and may have distinct biological consequences. Although limited by the representation of CpG sites on the Illumina array, which is biased towards genic regions and some enhancer/intergenic sites, but excludes repetitive DNA and other regions of constitutive heterochromatin, each radiation source affected the epigenome in distinct ways. For example, ^56^Fe ions have a propensity to affect regions of accessible chromatin such as gene promoters and distal regulatory elements (‘enhancers’) whereas ^28^Si ions preferentially affected DNA in more repressed, heterochromatic regions. Whether these differences in DNA methylation are a reflection of a difference in the susceptibilities of different chromatin regions to radiation-induced DNA damage or to its repair is unknown, but DNA damage (double strand breaks (DSB), oxidative damage) has been suggested to promote the recruitment of DNA methyltransferases and other histone modifiers (e.g. PRC1/2; SIRT1) to mediate local chromatin repression that persists in subsequent cell divisions^26,52–55^. Interestingly, an electron microscopic study of high-LET (carbon-ion) induced DNA damage showed that unlike X-irradiation, which induced DSBs that were distributed throughout the nucleus and efficiently cleared, carbon-ion irradiation induced clustered lesions along the particle trajectory that localized primarily to electron dense heterochromatic regions^56^. These phosphor-Ku70-bound clusters grew larger over time, suggesting inefficient repair. While there is no way to directly relate our nucleotide level analysis with these broader scale observations, it is intriguing to speculate that a persistence of radiation-induced lesions in heterochromatin might underlie the differences in tumor-promoting activities between high-LET and low-LET irradiation^57,58^ or even that between different HZE ions^47,49^.

Our results complement previous findings regarding DNA methylation content in high- and low-LET radiation exposed cells, and highlight the additional information provided by site-specific CpG methylation analyses. Many previous studies assessing the epigenomic impact of irradiation have focused on whole body irradiation in animals and analyses of bulk genomic 5mC levels and/or the methylation status of repetitive elements as a surrogate for global methylation levels. Using an array-based approach to assess site-specific methylation, our results are similar to those of Bae *et al*.^38^, who reported primarily losses of DNA methylation in colorectal cancer cells in response to gamma irradiation. Lahtz, *et al*.^59^ also observed a tendency towards hypomethylation, albeit modest, in normal human bronchial epithelial cells in response to gamma irradiation. Kim, *et al*.^40^, on the other hand observed a tendency for site-specific hypermethylation in breast cancer cells treated with particle (proton) irradiation with the same 450K array used here.

One limitation of our work is the inability to distinguish 5mC from 5hmC as both modifications are resistant to bisulfite-mediated conversion to uracil; the principle upon which the Illumina array-based method is based. Thus we cannot rule out the possibility that some of the observed alterations in DNA methylation may be due to changes in 5hmC. Significant changes to global and repetitive element 5hmC levels have been reported across various tissues, including mouse lung, in response to protons or various HZE ions (eg. ^56^Fe, ^28^Si. ^48^Ti)^27,28,33^, and recent genome-wide mapping studies have identified persistent site specific alterations in 5hmC in response to protons and/or ^56^Fe exposure^23–25^. As noted above, 5hmC is enzymatically derived from 5mC by the TET-family hydroxylases, and can be further oxidized to 5fC and 5caC. Though the function is incompletely understood, recent work suggests that 5hmC represents a distinct and stable epigenetic mark that accumulates in gene bodies and intragenic regions^14^. Indeed, the work of Impey *et al*.^24,25^ suggests that HZE-induced alterations in 5hmC arise from pre-existing 5mC sites. They observed a shift in the balance of 5mC to 5hmC across the bodies of tissue-relevant genes, suggesting that space irradiation induced alterations in 5hmC may be coupled to transcription. In contrast, 5fC and 5caC (but not 5hmC) preferentially accumulate in the regions surrounding CpG islands (‘shores’) and enhancer regions when base excision repair is compromised, suggesting that 5fC and 5caC are the primary intermediates in the DNA demethylation pathway and further, that these are the regions where the turnover of 5mC is the most dynamic^60–62^. We find here that the CpG sites affected by ^56^Fe were enriched in these regions, making it interesting to speculate that the increased methylation observed might reflect irradiation-induced impairment of 5mC turnover.

To probe the significance of our findings with respect to human lung cancer, we leveraged the human epigenome information available from hundreds of primary lung tumors that have been analyzed as part of the Cancer Genome Atlas (TCGA) project. We found that the methylation status of high-LET irradiation sensitive CpG sites, particularly those impacted by ^56^Fe ion exposure, could discriminate tumor from normal tissue for both lung adenocarcinomas and squamous cell lung carcinomas. No such relationship existed for the sites affected by ^28^Si ion or low-LET radiation exposure. Thus, our results suggest that HZE particle exposure creates a DNA methylation ‘signature’ that uniquely reflects cancer-specific methylation patterns observed in human primary lung cancers. Interestingly, a gene expression signature specifically associated with fractionated ^56^Fe ion -promoted lung tumorigenesis and derived 70 days after the initial insult in mouse lung could accurately predict overall survival among patients with lung or breast cancer^63^. That it is the ^56^Fe ion-affected signature in particular that is capable of segregating tumor from normal is perhaps not surprising given that the ^56^Fe ion-affected sites are enriched in the regions surrounding CpG islands (the ‘shores’) and in accessible regions with chromatin features indicative of weak/poised promoters and enhancers; regions of the genome that exhibit the most variable levels of methylation across tissues/cell types, between individuals^64–66^, and as noted above, represent areas of dynamic DNA methylation turnover. In contrast, CpGs within the CpG dense regions that encompass most promoters (CpG ‘islands’) typically remain unmethylated, and with few exceptions, maintain an open and permissive chromatin state (marked by H3K4me3; DNaseI hypersensitive) across tissues and cell types allowing for a wide-range of potential gene expression levels. Indeed, methylation of such regions is a relatively poor correlate of gene expression^67,68^. In contrast, CpG island ‘shores’ and enhancer elements exhibit the greatest variation in DNA methylation, and thus are better able to stratify normal tissues, cellular phenotypes, or patient outcomes^67–69^. While hypermethylation of normally unmethylated CpG island containing promoters is a well-described mechanism for the inactivation of tumor suppressor genes in cancer, recent studies underscore the contribution of altered methylation at enhancer elements as an important contributor to the aberrant gene expression programs that define human cancers^68,70,71^. Notably, a subset of the genes most closely linked to the ^56^Fe affected CpGs showed a significant overlap with genes previously determined to undergo promoter hypermethylation in primary human lung cancer^43^ where there was no overlap of the Si or X ray associated genes among this set (see Supplemental Table 2). Taken together, our data suggest that the imprint of a prior high-LET radiation exposure is reflected in the DNA methylation pattern, and may prove useful as a biomarker for long-term, individual cancer risk.

## Methods

### Cell Line and Culture conditions

The immortalized human bronchial epithelial cell line (HBEC3- KT) was established by introducing mouse Cdk4 and hTERT into normal human bronchial epithelial cells (HBECs)^41^ and were a kind gift from Dr. J.D. Minna of the University of Texas Southwestern Medical Center. Throughout this study the HBEC3-KT immortalized line was cultured in Serum-Free Keratinocyte Medium (K-SFM) supplemented with human recombinant Epithelial Growth Factor and Bovine Pituitary Extract (Life Technologies #17005-042). Triplicate biological replicates were irradiated and maintained independently. Cells were continually grown in a 5% CO_2_ environment at 37 °C and passaged (1:4) twice per week for three months. Cell pellets (1e6) were collected at each passage, flash frozen, and stored at −80 °C for subsequent DNA extraction.

### Irradiation

High-energy HZE particle irradiations were performed in Brookhaven, NY at the NASA Space Radiation Laboratory (NSRL). The X  ray (low-LET) exposures were conducted at Emory University using an X-RAD 320 biological irradiator (Precision X-Ray, North Branford, CT). Cells were shipped to BNL via overnight courier in T-75 culture flasks filled to capacity with growth media. Upon receipt, media levels were adjusted, and cells were allowed to recover for at least 24hrs. One day prior to irradiation, cells were trypsinized, counted, and seeded into T25 flasks allowing for biological triplicate flasks for each experimental condition including an unirradiated control. Three biological replicate cultures containing either 1 × 10^6^ cells (for acute time point) or 2 × 10^5^ cells (for continuous culture) in T-25 flasks were irradiated independently with 0, 0.1, 0.3 or 1.0 Gy ^56^Fe ions (Beam energy: 600 MeV/u; dose rate for the 0.1 Gy dose was 0.1 Gy/min, for the 0.3 Gy dose, 0.3 Gy/min, and for the 1.0 Gy dose, 1 Gy/min.) or with 0.0, 0.3, 1.0 Gy ^28^Si ions (Beam energy: 300 MeV/u; dose rate for the 0.3 Gy dose was 0.28 Gy/min, and for the 1.0 Gy dose, 0.63 Gy/min). Culture flasks were positioned orthogonally to the beam using an automated flipper provided at the NSRL. For each experiment, mock-irradiated controls (triplicate cultures) were seeded at the same time as the experimental flasks, from the same parent culture and handled identically (including travel to/from the NRSL facility), but were not placed in the beam line. Immediately following irradiation, all cultures were returned to a 37 °C incubator for forty-eight hours before cell pellets were collected from one set of flasks (triplicates, 2 day time point), while the remaining cultures were returned to the home laboratory at Emory University and maintained in continuous culture for an additional 3 months, with biweekly subculturing and DNA collection. X ray irradiations were performed using identical plating conditions but were exposed to doses of 0 Gy and 1.0 Gy (beam energy 320 kV; dose rate ~1 Gy/min) at Emory University. Cultures were maintained independently from the start of the experiment.

### DNA Methylation Profiling

Genomic DNA isolation was conducted at the time of sample processing for subsequent methylation analysis. Triplicate cell pellets, previously held at −80 °C, were processed using the All Prep DNA/RNA kit (Qiagen #80204) according to the manufacturer’s instructions. The methylation status of 485,577 CpG sites was then interrogated using the Illumina Infinium Human Methylation 450K platform (Illumina, San Diego, CA). DNA (1 μg) was bisulfite modified using the EZ DNA Methylation-Direct kit (Zymo Research #D5020), fragmented, amplified and hybridized to the Human Methylation 450K BeadChip according to the manufacturer’s instructions by the Emory Integrated Genomics Core Facility. All samples from a given exposure experiment were processed in parallel and on parallel chips with replicate samples randomized with respect to chip position.

### Differential DNA Methylation Analyses

A linear mixed effects model, CpGassoc^72^, was used to perform quality control and to identify DNA methylation changes significantly associated with dose, or with time after exposure. The ^56^Fe ion, ^28^Si ion, and X ray exposed cohorts were considered separately in the analyses, and each included 3 biological replicate samples for each of 24 doses and 4 time points per exposure type (^56^Fe ions, 4 doses × 4 time points = 48 samples; ^28^Si ions = 3 doses × 4 time points = 27 samples; X ray, 2 doses × 4 time points = 24 samples). For each CpG site, the signals from methylated (M) and unmethylated (U) bead types were quantile normalized together (using the R-package limma^73^; and then used to calculate β-values [β = M/(U + M)], which approximate the proportion of DNA methylated at each CpG. Data points with detection p-values > 0.001 were set to missing, and CpG sites with missing data for >10% of samples were excluded from the analysis. Samples with a probe detection call rate <95%, or an average signal intensity <2,000 AU or <50% of the experiment-wide sample median were also excluded. This resulted in a total of 484,434 (^56^Fe ion); 484,765 (^28^Si ion) and 484,384 (X ray) CpGs considered in the subsequent analysis. A linear mixed effects model was applied to identify DNA methylation changes significantly correlated with dose. Intra-experiment β-values were modeled as a linear function of radiation dose, with covariates adjusting for time-after-exposure, row on chip, and a random effect for chip number. The Holm (step-down Bonferroni) method was applied to correct for multiple comparisons^74^. CpG sites with a corrected p-value < 0.05 were considered nominally significant and CpGs with an uncorrected p-value < 0.001 were considered moderately significant (Supplemental Table 1). To identify methylation changes associated with time-after-exposure independent of dose, the same strategy was used to model β-values as a function of time-after-exposure, with covariates adjusting for radiation dose, and assay variables.

### Genomic Annotation and Meta-Analyses

CpGs were annotated to the nearest UCSC CpG Island (CGI) and RefSeq (v.75) transcription start site (TSS) using custom R/Bioconductor scripts^75^. CpGs were categorized into those overlapping a CGI, or those within 2 kb upstream (5′Shore), 2 kb downstream (3′Shore), or between the downstream shore and the transcription termination site (Gene Body). CpG sites not falling into one of these classes were considered as ‘other/intergenic’. The distribution of all CpGs covered by the assay versus those determined to be hypermethylated or hypomethylated were plotted relative to the nearest CGI or TSS using the density function in R/Bioconductor, where the width of the CGI was scaled to the average width of UCSC CGIs.

ENCODE^76^ ChIP-seq data sets derived from A549 lung cancer cells (H3K27Ac, ENCSR000AUI; H3K4me3 ENCSR000ASH; DNaseI, ENCSR136DNA) were downloaded as mapped bam files from the ENCODE project website (https://www.encodeproject.org). The average tag densities surrounding each set of CpG sites were calculated in 20 bp bins using the GenomicRanges R package^77^, and normalized to the total number of mapped reads.

ChromHMM^42^ chromatin state maps derived from normal human mammary epithelial cells (ENCFF687QKV) were used to annotate each CpG site to a chromatin compartment. For clarity, both ‘Strong Enhancer’ (states 4 and 5) and both ‘Weak Enhancer’ (states 6 and 7) were merged. States 9, 10, and 11 (‘Transcriptional Transition’, ‘Transcriptional Elongation’, and ‘Weak Transcription’) were merged and referred to as ‘Transcribed Regions’. Odds ratios were calculated based on the number of affected sites in each compartment vs. the distribution of the CpGs on the array as a whole using Fisher’s Exact test.

For functional gene annotation analyses, CpGs significantly associated with radiation dose were mapped to the closest UCSC hg19 KnownGene transcript using the ‘GenomicRanges’ (v1.30.0) and ‘TxDb.Hsapiens.UCSC.hg19.knownGene’ (v3.2.2) packages in R/Bioconductor (v3.4.3) to construct input gene sets (symbols). Gene sets corresponding to CpGs altered for each radiation type were analyzed for overlap (hypergeometric distribution) with the MSigDB Hallmark (H), Curated (C2), Computational (C4), Gene Ontology Molecular Function (MF), Oncogenic (C6) and Immunologic (C7) data sets using the Gene Set Enrichment Analysis web interface (v6.1) available at http://www.broadinstitute.org/gsea/msigdb/ ^78^ and for functional annotation clustering using the DAVID Bioinformatics Resource (http://david.abcc.ncifcrf.gov)^79^ using an EASE of 0.05 and the UP_KEYWORDS; GO_BP_DIRECT; GO_MF_DIRECT; KEGG_PATHWAYS; and DOMAIN_INTERPRO data sets.

### Analysis of Lung Cancer TCGA Data

Illumina Infinium HumanMethylation 450 K methylation data for the ^56^Fe ion- (n = 935), ^28^Si ion- (n = 300) and X ray- (n = 1150) affected CpG sites was extracted for 25 matched tumor normal pairs of lung adenocarcinoma (LUAD) and lung squamous cell carcinoma (LUSC) from patients identified through the TCGA data portal (https://tcga-data.nci.nih.gov/tcga/). CpG probes with a detection p-value > 0.05 across the sample set were excluded, leaving 784, 938, and 237 CpG sites from the ^56^Fe ion-, ^28^Si ion-, and X ray-affected sites, respectively. The methylation levels (beta values) from these sites were then used in an unsupervised, hierarchical cluster analysis based on the Manhattan distance and agglomerative complete linkage. The statistical significance of each set of exposure associated CpG sites in separating lung tumors from normal was assessed versus a randomly selected set of CpG sites of the same number using a bootstrap approach. Specifically, we randomly sampled M = 1,000 times the same number of CpG sites in each exposure set from among a total of 391,954 (LUAD) or 395,241 (LUSC) CpG sites on the array that remained after exclusion of low quality probes (detection p-value across all samples >0.05). For each resampling, dendrograms were constructed using the same unsupervised clustering approach and cut based on a fixed number of k = 2 clusters. An association analysis was performed based on a Chi-Square test for each resampling and p-values obtained. A Monte Carlo p-value was used to compare the ability of randomly-sampled CpG sites to separate tumor and normal samples into two clusters versus the p-value obtained from the CpG sites defined by each group (^56^Fe, ^28^Si, X ray).

### Data availability

The ChIP-seq and DNAse-seq datasets used in the analyses are available in the ENCODE repository, https://www.encodeproject.org under the accession numbers noted above. DNA methylation array data generated as part of the current study has been deposited in the GEO repository (https://www.ncbi.nlm.nih.gov/geo/) under the accession number GSE108187.

## Electronic supplementary material

Supplemental Data

Supplemental Table 1

Supplemental Table 2

## References

[1] Held KD (2009). Effects of low fluences of radiations found in space on cellular systems. Int. J. Radiat. Biol..

[2] Hu W (2014). Effects of shielding on the induction of 53BP1 foci and micronuclei after Fe ion exposures. J. Radiat. Res..

[3] Lebel EA (2011). Analyses of the Secondary Particle Radiation and the DNA Damage It Causes to Human Keratinocytes. J. Radiat. Res..

[4] Mukherjee B, Camacho CV, Tomimatsu N, Miller J, Burma S (2008). Modulation of the DNA-damage response to HZE particles by shielding. DNA Repair (Amst)..

[5] Durante M, Cucinotta FA (2008). Heavy ion carcinogenesis and human space exploration. Nat. Rev. Cancer.

[6] Cucinotta FA, Durante M (2006). Cancer risk from exposure to galactic cosmic rays: implications for space exploration by human beings. Lancet Oncol..

[7] Plante I, Ponomarev AL, Cucinotta FA (2013). Calculation of the energy deposition in nanovolumes by protons and HZE particles: geometric patterns of initial distributions of DNA repair foci. Phys. Med. Biol..

[8] Cucinotta FA, Cacao E (2017). Non-Targeted Effects Models Predict Significantly Higher Mars Mission Cancer Risk than Targeted Effects Models. Sci. Rep..

[9] Shinoto M, Ebner DK, Yamada S (2016). Particle Radiation Therapy for Gastrointestinal Cancers. Curr. Oncol. Rep..

[10] Ding L-H (2013). Distinct transcriptome profiles identified in normal human bronchial epithelial cells after exposure to γ-rays and different elemental particles of high Z and energy. BMC Genomics.

[11] Huidobro C, Fernandez AF, Fraga MF (2013). Aging epigenetics: causes and consequences. Mol. Aspects Med..

[12] Jones PA (2012). Functions of DNA methylation: islands, start sites, gene bodies and beyond. Nat. Rev. Genet..

[13] Avvakumov GV (2008). Structural basis for recognition of hemi-methylated DNA by the SRA domain of human UHRF1. Nature.

[14] Rasmussen KD, Helin K (2016). Role of TET enzymes in DNA methylation, development, and cancer. Genes Dev..

[15] Hashimoto H, Zhang X, Vertino PM, Cheng X (2015). The mechanisms of generation, recognition, and erasure of DNA 5-methylcytosine and thymine oxidation. Journal of Biological Chemistry.

[16] Miousse, I. R. *et al*. Inter-Strain Differences in LINE-1 DNA Methylation in the Mouse Hematopoietic System in Response to Exposure to Ionizing Radiation. *Int. J. Mol. Sci*. **18** (2017). 10.3390/ijms18071430 PMC5535921 28677663

[17] Miousse IR (2014). Exposure to low-dose (56)Fe-ion radiation induces long-term epigenetic alterations in mouse bone marrow hematopoietic progenitor and stem cells. Radiat. Res..

[18] Koturbash I (2016). Radiation-induced changes in DNA methylation of repetitive elements in the mouse heart. Mutat. Res..

[19] Wang J (2014). Genome-wide screen of DNA methylation changes induced by low dose X-ray radiation in mice. PLoS One.

[20] Newman MR (2014). The methylation of DNA repeat elements is sex-dependent and temporally different in response to X radiation in radiosensitive and radioresistant mouse strains. Radiat. Res..

[21] Prior S (2016). Densely ionizing radiation affects DNA methylation of selective LINE-1 elements. Environ. Res..

[22] Nzabarushimana E (2014). Long-term epigenetic effects of exposure to low doses of 56Fe in the mouse lung. J. Radiat. Res..

[23] Impey S (2016). Proton irradiation induces persistent and tissue-specific DNA methylation changes in the left ventricle and hippocampus. BMC Genomics.

[24] Impey, S. *et al*. Short- and long-term effects of 56Fe irradiation on cognition and hippocampal DNA methylation and gene expression. *BMC Genomics* **17** (2016). 10.1186/s12864-016-3110-7 PMC5078898 27776477

[25] Impey S (2017). Bi-directional and shared epigenomic signatures following proton and (56)Fe irradiation. Sci. Rep..

[26] Acharya, M. M. *et al*. Epigenetic determinants of space radiation-induced cognitive dysfunction. *Sci. Rep*. **7** (2017). 10.1038/srep42885 PMC5318883 28220892

[27] Nzabarushimana E (2015). Combined exposure to protons and (56)Fe leads to overexpression of Il13 and reactivation of repetitive elements in the mouse lung. Life Sci. Sp. Res..

[28] Jangiam W, Tungjai M, Rithidech KN (2015). Induction of chronic oxidative stress, chronic inflammation and aberrant patterns of DNA methylation in the liver of titanium-exposed CBA/CaJ mice. Int. J. Radiat. Biol..

[29] Rithidech KN (2016). Induction of Chronic Inflammation and Altered Levels of DNA Hydroxymethylation in Somatic and Germinal Tissues of CBA/CaJ Mice Exposed to (48)Ti Ions. Front. Oncol..

[30] Ilnytskyy Y, Koturbash I, Kovalchuk O (2009). Radiation-induced bystander effects *in vivo* are epigenetically regulated in a tissue-specific manner. Environ. Mol. Mutagen..

[31] Pogribny I, Raiche J, Slovack M, Kovalchuk O (2004). Dose-dependence, sex- and tissue-specificity, and persistence of radiation-induced genomic DNA methylation changes. Biochem. Biophys. Res. Commun..

[32] Filkowski JN (2010). Hypomethylation and genome instability in the germline of exposed parents and their progeny is associated with altered miRNA expression. Carcinogenesis.

[33] Lima F, Ding D, Goetz W, Yang AJ, Baulch JE (2014). High LET 56Fe ion irradiation induces tissue-specific changes in DNA methylation in the mouse. Environ. Mol. Mutagen..

[34] Rithidech KN (2015). Late-occurring chromosome aberrations and global DNA methylation in hematopoietic stem/progenitor cells of CBA/CaJ mice exposed to silicon ((28)Si) ions. Mutat. Res..

[35] Aypar U, Morgan WF, Baulch JE (2011). Radiation-induced epigenetic alterations after low and high LET irradiations. Mutat. Res. Mol. Mech. Mutagen..

[36] Antwih DA, Gabbara KM, Lancaster WD, Ruden DM, Zielske SP (2013). Radiation-induced epigenetic DNA methylation modification of radiation-response pathways. Epigenetics.

[37] Kovalchuk O (2004). Methylation changes in muscle and liver tissues of male and female mice exposed to acute and chronic low-dose X-ray-irradiation. Mutat. Res..

[38] Bae J-H (2015). Identification of radiation-induced aberrant hypomethylation in colon cancer. BMC Genomics.

[39] Goetz W, Morgan MNM, Baulch JE (2011). The effect of radiation quality on genomic DNA methylation profiles in irradiated human cell lines. Radiat. Res..

[40] Kim B (2016). Proton Beams Inhibit Proliferation of Breast Cancer Cells by Altering DNA Methylation Status. J. Cancer.

[41] Ramirez RD (2004). Immortalization of human bronchial epithelial cells in the absence of viral oncoproteins. Cancer Res..

[42] Ernst J, Kellis M (2012). ChromHMM: automating chromatin-state discovery and characterization. Nat. Methods.

[43] Fukasawa M (2006). Microarray analysis of promoter methylation in lung cancers. J. Hum. Genet..

[44] Cardis E (2007). The 15-Country Collaborative Study of Cancer Risk among Radiation Workers in the Nuclear Industry: estimates of radiation-related cancer risks. Radiat. Res..

[45] Preston DL (2007). Solid cancer incidence in atomic bomb survivors: 1958-1998. Radiat. Res..

[46] Cucinotta FA (2014). Space Radiation Risks for Astronauts on Multiple International Space Station Missions. PLoS One.

[47] Suman S (2016). Relative Biological Effectiveness of Energetic Heavy Ions for Intestinal Tumorigenesis Shows Male Preponderance and Radiation Type and Energy Dependence in APC(1638N/+) Mice. Int. J. Radiat. Oncol. Biol. Phys..

[48] Trani D, Datta K, Doiron K, Kallakury B, Fornace AJ (2010). Enhanced Intestinal Tumor Multiplicity and Grade *in vivo* after HZE Exposure: Mouse Models for Space Radiation Risk Estimates. Radiat. Environ. Biophys..

[49] Wang X (2015). Relative Effectiveness at 1 Gy after Acute and Fractionated Exposures of Heavy Ions with Different Linear Energy Transfer for Lung Tumorigenesis. Radiat. Res..

[50] Weil MM (2009). Incidence of acute myeloid leukemia and hepatocellular carcinoma in mice irradiated with 1 GeV/nucleon (56)Fe ions. Radiat. Res..

[51] Cucinotta FA, Chappell LJ (2011). Updates to astronaut radiation limits: radiation risks for never-smokers. Radiat. Res..

[52] Ding N (2016). Mismatch repair proteins recruit DNA methyltransferase 1 to sites of oxidative DNA damage. J. Mol. Cell Biol..

[53] O’Hagan HM (2014). Chromatin modifications during repair of environmental exposure-induced DNA damage: a potential mechanism for stable epigenetic alterations. Environ. Mol. Mutagen..

[54] O’Hagan HM, Mohammad HP, Baylin SB (2008). Double strand breaks can initiate gene silencing and SIRT1-dependent onset of DNA methylation in an exogenous promoter CpG island. PLoS Genet..

[55] O’Hagan HM (2011). Oxidative damage targets complexes containing DNA methyltransferases, SIRT1, and polycomb members to promoter CpG Islands. Cancer Cell.

[56] Lorat Y, Timm S, Jakob B, Taucher-Scholz G, Rübe CE (2016). Clustered double-strand breaks in heterochromatin perturb DNA repair after high linear energy transfer irradiation. Radiother. Oncol. J. Eur. Soc. Ther. Radiol. Oncol..

[57] Bielefeldt-Ohmann H, Genik PC, Fallgren CM, Ullrich RL, Weil MM (2012). Animal studies of charged particle-induced carcinogenesis. Health Phys..

[58] Datta K, Suman S, Kallakury BVS, Fornace AJ (2013). Heavy ion radiation exposure triggered higher intestinal tumor frequency and greater β-catenin activation than γ radiation in APC(Min/+) mice. PLoS One.

[59] Lahtz C (2012). Gamma Irradiation Does Not Induce Detectable Changes in DNA Methylation Directly following Exposure of Human Cells. PLoS One.

[60] Shen L (2013). Genome-wide analysis reveals TET- and TDG-dependent 5-methylcytosine oxidation dynamics. Cell.

[61] Song CX (2013). Genome-wide profiling of 5-formylcytosine reveals its roles in epigenetic priming. Cell.

[62] Wu H, Wu X, Shen L, Zhang Y (2014). Single-base resolution analysis of active DNA demethylation using methylase-assisted bisulfite sequencing. Nat. Biotechnol..

[63] Delgado O (2014). Radiation-enhanced Lung Cancer Progression in a Transgenic Mouse Model of Lung Cancer is Predictive of Outcomes in Human Lung and Breast Cancer. Clin. Cancer Res..

[64] Irizarry RA (2009). The human colon cancer methylome shows similar hypo- and hypermethylation at conserved tissue-specific CpG island shores. Nat. Genet..

[65] Kundaje A (2015). Integrative analysis of 111 reference human epigenomes. Nature.

[66] Ziller MJ (2013). Charting a dynamic DNA methylation landscape of the human genome. Nature.

[67] Aran D, Hellman A (2014). Unmasking risk loci: DNA methylation illuminates the biology of cancer predisposition: analyzing DNA methylation of transcriptional enhancers reveals missed regulatory links between cancer risk loci and genes. BioEssays News Rev. Mol. Cell. Dev. Biol..

[68] Aran D, Sabato S, Hellman A (2013). DNA methylation of distal regulatory sites characterizes dysregulation of cancer genes. Genome Biol..

[69] Wiench M (2011). DNA methylation status predicts cell type-specific enhancer activity. EMBO J..

[70] Bell RE (2016). Enhancer methylation dynamics contribute to cancer plasticity and patient mortality. Genome Res..

[71] Taberlay PC, Statham AL, Kelly TK, Clark SJ, Jones PA (2014). Reconfiguration of nucleosome depleted regions at distal regulatory elements accompanies DNA methylation of enhancers and insulators in cancer. Genome Res.

[72] Barfield RT, Kilaru V, Smith AK, Conneely KN (2012). CpGassoc: an R function for analysis of DNA methylation microarray data. Bioinformatics.

[73] Smyth, G. K. limma: Linear Models for Microarray Data. In *Bioinformatics and Computational Biology Solutions* Using R *and Bioconductor* (eds Gentleman, R., Carey, V. J., Huber, W., Irizarry, R. A. & Dudoit, S.) 397–420 (Springer New York, 2005).

[74] Holm S (1979). A Simple Sequentially Rejective Multiple Test Procedure. Scand. J. Stat..

[75] Gentleman RC (2004). Bioconductor: open software development for computational biology and bioinformatics. Genome Biol..

[76] An integrated encyclopedia of DNA elements in the human genome. *Nature* **489**, 57–74 (2012). 10.1038/nature11247 PMC3439153 22955616

[77] Lawrence M (2013). Software for Computing and Annotating Genomic Ranges. PLoS Comput Biol.

[78] Subramanian A (2005). Gene set enrichment analysis: A knowledge-based approach for interpreting genome-wide expression profiles. Proc. Natl. Acad. Sci. USA.

[79] Dennis G (2003). DAVID: Database for Annotation, Visualization, and Integrated Discovery. Genome Biol..

