# Supplementary material for: Galactic Cosmic Radiation Induces Persistent Epigenome Alterations Relevant to Human Lung Cancer
Source: Sci Rep. 2018 Apr 30;8:6709. doi: 10.1038/s41598-018-24755-8 (PMC5928241; doi:10.1038/s41598-018-24755-8)
Supplement: Supplementary file 1 — Supplemental Data [file 41598_2018_24755_MOESM1_ESM.pdf]

SUPPLEMENTAL DATA:

GALACTIC COSMIC RADIATION INDUCES PERSISTENT EPIGENOME ALTERATIONS RELEVANT TO HUMAN LUNG CANCER

E.M. Kennedy[1,2#], D.R. Powell[3#], Z. Li[4], J.S.K. Bell[1,3†], B.G. Barwick[3], H. Feng[2], M.R. McCrary[3], B. Dwivedi[5], J. Kowalski[5,6], W.S. Dynan[3,4,6], K.N. Conneely[1,2,5] and P.M. Vertino[3,6]*

[1]Graduate Program in Genetics and Molecular Biology, Departments of [2]Human Genetics, [3]Radiation Oncology, and [4]Biochemistry, Emory University School of Medicine; [5]Department of Biostatistics and Bioinformatics, Rollins School of Public Health, Emory University; and [6]the Winship Cancer Institute of Emory University, Atlanta, GA 30322

Supplemental Figure 1. Time-dependent methylation drift

a) Comparison of the average methylation (β-value) of each CpG site in the X ray-exposed cohort at the indicated time-after-exposure relative to that on Day 2. Red indicates those CpGs that are significantly hypermethylated with time (n=2,294); green are those sites significantly hypomethylated with time (n=647; $p<1e\text{-}7$). **b)** A linear mixed effects model was applied to identify DNA methylation changes significantly associated with time-after-exposure for each exposure type. Shown are scatter plots comparing the significance and direction of change (t-statistics) for CpG sites at which methylation changed significantly with time-after-exposure in the ^{28}Si exposed series, the ^{56}Fe exposed series, and the X ray exposed series. A positive t-statistic indicates a gain in methylation (hypermethylated) and a negative t-statistic indicates a loss of methylation (hypomethylation). Light green indicates sites reaching an FDR<0.05 (Benjamini-Hochberg) and blue are those reaching Holm significance ($p<1e\text{-}7$). **c)** Venn diagrams comparing the overlap of CpG sites whose methylation change was significantly associated with time in culture [FDR<0.05 (Benjamini-Hochberg)] in the ^{28}Si exposed series, the ^{56}Fe exposed series, and the X ray exposed series. **d)** Average methylation level (β) over time among CpG sites negatively (left) or positively (right) associated with time stratified by ^{56}Fe-ion dose. Analysis was restricted to those CpG sites found to be independently associated with both dose and time in the ^{56}Fe-ion exposed series (n=91).

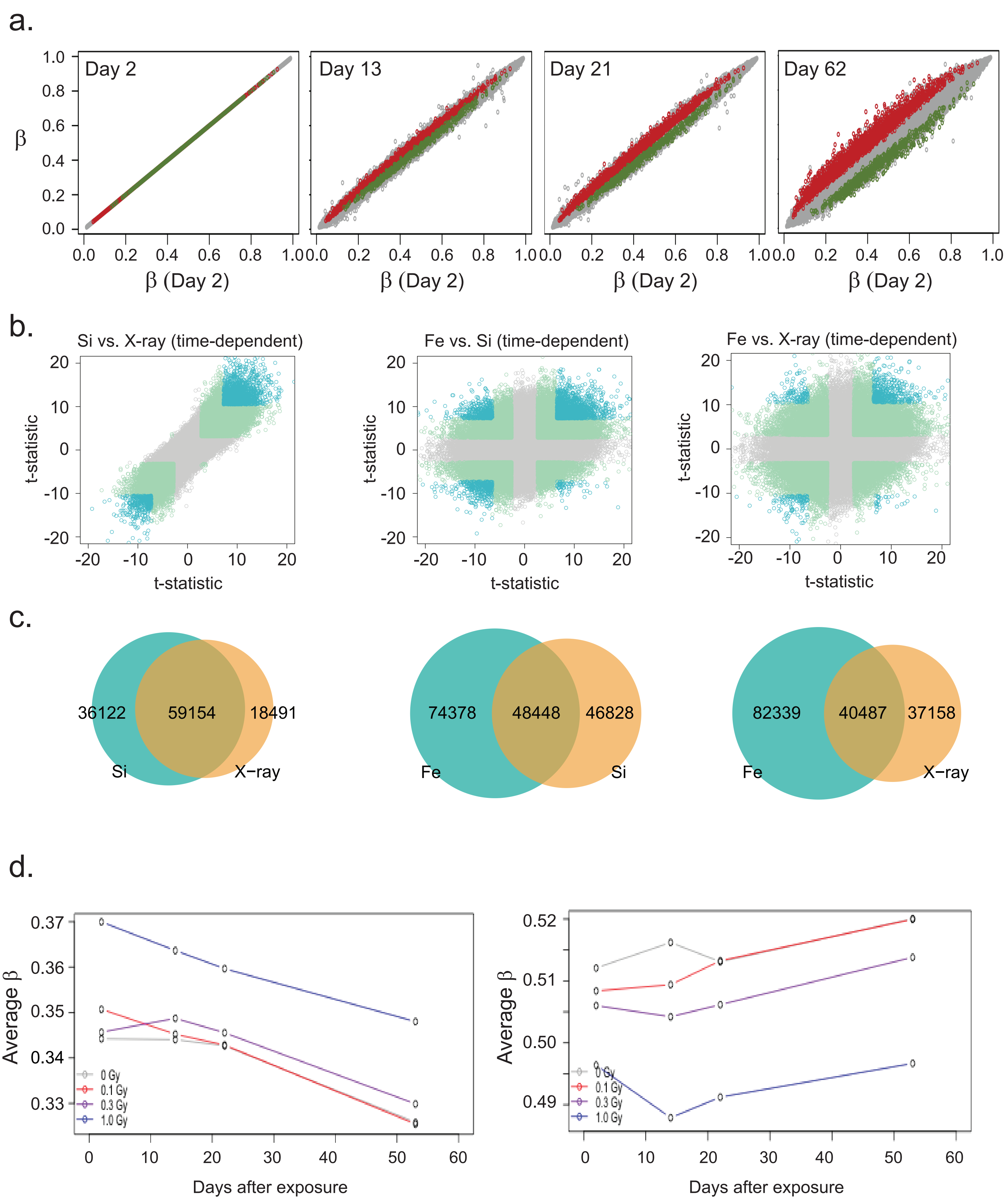

a.
Day 2
Day 13
Day 21
Day 62
β
β (Day 2)
b.
Si vs. X-ray (time-dependent)
Fe vs. Si (time-dependent)
Fe vs. X-ray (time-dependent)
t-statistic
c.
36122
59154
18491
Si
X−ray
74378
48448
46828
Fe
Si
82339
40487
37158
Fe
X−ray
d.
Average β
Days after exposure
0 Gy
0.1 Gy
0.3 Gy
1.0 Gy
